# *MIF rs755622* and *IL6 rs1800795* Are Implied in Genetic Susceptibility to End-Stage Renal Disease (ESRD)

**DOI:** 10.3390/genes13020226

**Published:** 2022-01-25

**Authors:** Marco Guarneri, Letizia Scola, Rosa Maria Giarratana, Manuela Bova, Caterina Carollo, Loredana Vaccarino, Leonardo Calandra, Domenico Lio, Carmela Rita Balistreri, Santina Cottone

**Affiliations:** 1 Unit of Nephrology & Hypertension, European Society of Hypertension Excellence Center, Department of Health Promotion Sciences, Maternal & Infant Care, Internal Medicine & Medical Specialties (PROMISE), University of Palermo, “Paolo Giaccone” University Hospital, 90127 Palermo, Italy; marco.guarneri@unipa.it (M.G.); caterina.carollo@unipa.it (C.C.); leonardo.calandra@unipa.it (L.C.); santina.cottone@unipa.it (S.C.); 2 Clinical Pathology, Department of Bio-Medicine, Neuroscience, and Advanced Diagnostics, University of Palermo, 90100 Palermo, Italy; letizia.scola@unipa.it (L.S.); rosamaria.giarratana@unipa.it (R.M.G.); manuela.bova@unipa.it (M.B.); loredana.vaccarino@unipa.it (L.V.); carmelarita.balistreri@unipa.it (C.R.B.)

**Keywords:** MIF, IL6, SNP, CKD, ESRD

## Abstract

Chronic kidney disease (CKD) is characterized by an increased risk of kidney failure and end-stage renal disease (ESRD). Aging and comorbidities as cardiovascular diseases, metabolic disorders, infectious diseases, or tumors, might increase the risk of dialysis. In addition, genetic susceptibility factors might modulate kidney damage evolution. We have analyzed, in a group of ESRD patients and matched controls, a set of SNPs of genes (*Klotho rs577912*, *rs564481*, *rs9536314*; *FGF23 rs7955866*; *IGF1 rs35767*; *TNFA rs1800629*; *IL6 rs1800795*; *MIF rs755622*, *rs1007888*) chosen in relation to their possible involvement with renal disease and concomitant pathologies. Analysis of the raw data did indicate that *IL6 rs180795* and *MIF rs755622* SNPs might be markers of genetic susceptibility to ESRD. In particular, the C positive genotypes of *MIF rs755622*, (dominant model) seem to be an independent risk factor for ESDR patients (data adjusted for age, gender, and associated pathologies). Stratifying results according to age *MIF rs755622* C positive genotype frequencies are increased in both the two age classes considered (<59 and ≥59-year-old subjects). Analyses of data according to gender allowed us to observe that ESRD women shoved a significantly reduced frequency of genotypes bearing *IL6 rs180795* C allele. In addition, *MIF rs755622* might interact with diabetes or hypercholesterolemia in increasing susceptibility to ESRD. In conclusion, our data indicate that some polymorphisms involved in the regulation of both renal function and inflammatory response can influence the evolution of chronic kidney disease and suggest that the modulation of the activities of these and other genes should also be considered as therapeutic targets on to intervene with innovative therapies.

## 1. Introduction

Chronic kidney disease (CKD) is characterized by a progressive decrease of renal function with an increased risk of kidney failure and end-stage renal disease (ESRD), the disease stage where dialysis and transplantation are needed [1]. The National Kidney Foundation’s Kidney Disease Outcomes Quality Initiative (K/DOQI) has defined the stage of chronic kidney disease (CKD) based on the value of glomerular filtration rate (GFR) [2]. According to this classification, a GFR ≤ 59 mL/min/1.73 m^2^ or less defines a moderate to severe decrease in renal function.

In recent years, many studies have been carried out aimed at identifying the molecules to be used as the most sensitive biomarkers of kidney function, many of which are proteins produced by different segments of the nephrons, cytokines, receptors that might be implied in the kidney acute or chronic damage [3,4]. In addition, genes coding for these mediators are highly polymorphic and the effect of their interactions on the pathogeneses of different diseases might be influenced by the different individual genetic backgrounds [5].

Recently, the scenario of possible biomarkers in chronic renal failure has been enriched by new players such as Fibroblast Growth Factor-23 (FGF-23) and Klotho. The progressive deterioration of renal function is characterized by a progressive reduction of Klotho protein and an increase of the FGF-23 levels [6,7,8].

Klotho has anti-inflammatory and anti-fibrotic activity, preventing vascular calcifications. In humans, the circulating levels of Klotho decrease with age in serum, and polymorphisms of the *Klotho* gene related to premature aging have also been identified [9,10].

Circulating Klotho can be considered an early diagnostic marker of CKD. Already for estimated glomerular filtration rate (eGFR) values below 90 mL/min, there is a progressive reduction. Clinical and experimental studies have shown that a significant decrease in *Klotho* expression in the kidney has a direct correlation with the reduction of GFR-23 in CKD subjects [11]. High levels of FGF-23 and reduction of circulating Klotho levels in CKD correlate with a variety of clinical conditions such as left ventricular hypertrophy, vascular calcifications, and even an increased risk of mortality [12] in CKD patients. In this scenario, the SNPs of these genes can play a role.

In particular, *rs577912* polymorphism of the *Klotho* gene sequence might affect the expression of the protein and therefore its serum levels. The CC genotype is associated with a 16–21% lower *Klotho* expression compared to the AA/AC genotype with an association with an increased risk of mortality at 1 year from the start of dialysis [13]. In addition, the SNP *rs7955866* in exon 3 of *FGF23* has been related to renal damage [14]. The minor allele of this SNP induces a missense mutation (T239M) with a conformational change in the beta-sheets of the protein. Human embryonic kidney cells transfected in vitro with the *FGF23-239M* plasmid release higher concentrations of FGF-23 [14]. On the other hand, *Klotho* polymorphisms involved in metabolic disorders and cardiovascular pathologies might play a role in favoring progression to ESRD. In particular, *rs564481* and *rs9536314* might play a role, being implied in metabolic diseases and potentially influencing the prognosis of CKD [15,16,17,18]. In particular, *rs56481* might influence hypertension susceptibility and creatinine serum levels in different populations [15,16,17] whereas *rs9536314* might influence the kidney failure susceptibility impinging on the kidney function decline due to aging. Actually, a pathophysiological modification associated with chronic kidney disease mimics that of elderly individuals, which underlies shared clinical characteristics such as an increased risk of infection and atherosclerosis [18,19]. Moreover, *rs9536314* was the polymorphism most frequently involved in association studies on cardiovascular and non-cardiovascular mortality. This may be particularly relevant in patients with chronic kidney disease who have decreased Klotho levels in tissues and blood [20].

More recently, the interactions between the Klotho/FGF-23 system and the growth hormone (GH)/insulin-like growth factor-1 (IGF-1) axis have been analyzed. The GH/IGF-1 system appears relevant for glomerular and tubular function, as well as the synthesis of Klotho [21]. On the other hand, beneficial effects of IGF-1 in CKD are antagonized by Suppressor of Cytokine Signaling, due to CKD-associated chronic inflammation that might be responsible for GH insensitivity observed [22] and could be modulated by SNPs of *IGF1* gene [23,24]. Moreover, as reported in a recent meta-analysis [25], *IGF1 rs35767* increases the risk of renal disease.

Actually, the chronic inflammation state that characterizes CKD and ESRD induces an up-regulation of proinflammatory cytokines and the associated biomarkers (interleukin (IL)-1β, IL-1 receptor antagonist, IL-6, tumor necrosis factor (TNF)-α, C-reactive protein, and fibrinogen) [26,27].

Proinflammatory markers are shown to be important in the development of atherosclerotic complications and inflammation. In addition, it has been associated with hypertension in both experimental animal models and human studies [28,29,30,31,32], however, due to the complexity of this relationship, the mechanisms between inflammation and vascular involvement are still unclear.

On the other hand, an aging kidney can be considered a paradigmatic model of immunosenescence characterized by defective immune responses and increased systemic inflammation. In the kidney, resident macrophages and fibroblasts are continuously exposed to environmental stimuli, and the effects of cellular reprogramming induced by local immune responses, which accumulate with age, might have a role in the increased susceptibility to kidney disease among elderly individuals [19,33] and premature aging [34]. Key proinflammatory cytokines have been analyzed by different research groups both as circulating markers and as components of the genetic background predisposing to CKD. Cytokines of the TNF family [35,36,37], *TNF* SNPs [38,39], IL-6 serum levels [40,41], and *IL6* gene SNPs [42,43,44], as well as macrophage inhibiting factor (MIF) and *MIF* gene variants [45,46,47,48], might play a role in favoring kidney damage and have been studied. In particular, the *rs1800629* and *rs1800795* polymorphisms of *TNFA* and *IL6* genes can selectively affect spontaneous production of inflammatory cytokines influencing kidney function decrease and consequent development and progression of chronic kidney disease.

Herein, data on some genetic variants of key cytokine, mediator, and receptors involved in the complex scenario of kidney chronic failure have been reported and their relevance as risk profile markers useful in the prediction of kidney disease development and progression have been evaluated.

## 2. Materials and Methods

### 2.1. Patients and Controls

In collaboration with the Department of Nephrology and Dialysis with C.R.R. for arterial hypertension, “Paolo Giaccone” University Hospital, a population of 93 patients with end-stage renal chronic failure (ESRD) who were routinely undergoing dialysis therapy was recruited. Two control populations, for a total of 213 individuals, were recruited, one of healthy young people and one of the people over 59 years old, at the Department of Medical Biotechnology of the University of Palermo and in collaboration with the U.O.C of Transfusion Medicine of the Paolo Giaccone” University Hospital. The cut-off for age between the two control population was chosen on the basis of the median age of ESRD patients. The ≥59-year-old control population included both healthy subjects and people with hypercholesterolemia, type 2 diabetes, or hypertension without renal impairment (glomerular filtration rate, GFR ≥ 90 mL/min). Persons with autoimmune or tumor pathologies or with GFR < 90 were excluded from the control group. The clinical and demographic characteristics of the populations studied are shown in Table 1.

Our study was performed in accordance with ethical standards of the Helsinki Declaration of the World Medical Association and Italian legislation, and was approved by the local institutional review board (Comitato Etico Palermo 1, protocol code CET1 04/2020, date of approval 22 April 2020). All participants gave their informed consent and the data has been encoded to ensure the protection of patient privacy and controls. The EDTA peripheral blood samples used for molecular typing respectively were collected and stored at 70 °C until their use.

### 2.2. SNP Genotyping

DNA were extracted using a salting-out protocol and DNA quality was verified by 260/280 nm absorbance ratio and gel electrophoresis as previously reported [49]. SNP typing was performed using an on-demand assay developed by KBioscience Ltd. (Middlesex, UK) and based on homogeneous FRET (Fluorescence Resonance Energy Transfer) analysis on the products of a specific allele PCR (Kaspar) [50,51]. SNPs are listed in Table 2. Information on these polymorphisms was acquired from dbSNP NCBI (https://www.ncbi.nlm.nih.gov/snp/, accessed on 18 January 2022).

### 2.3. Statistical Analysis

Allele and genotype frequencies were evaluated by gene count. Pearson’s test was applied to test the Hardy–Weinberg equilibrium. Power of calculation has been evaluated for all comparisons made using an online tool (https://sample-size.net/, accessed on 17 February 2022). Significant differences in genotype distributions between groups were calculated using chi-square or Fisher’s exact test applied to the appropriated 2 × 2 or 3 × 2 contingency tables. Multiple logistic regression models were applied using overdominant (homozygous major allele and/or homozygous minor allele genotypes versus heterozygous genotype), codominant (heterozygous genotype versus homozygous major allele plus homozygous minor allele genotypes), dominant (homozygous plus heterozygous minor allele genotypes versus homozygous major allele genotype), and recessive (homozygous minor allele genotype versus homozygous major allele plus heterozygous genotypes) of heredity and adjusting the results for age, sex, and concomitance of existing pathologies. The choice of the most suitable model was based on the Bayesian Information Criterion (BIC) and the Akaike Information Verification Test (AIC). Odds ratio (OR) values and 95% confidence intervals (95% CI) were calculated and a *p*-value < 0.05 was considered statistically significant. Statistical analyzes were conducted using version 3.06 of GraphPadInStat software (GraphPad, San Diego, CA, USA) and online statistical analysis tools (https://www.snpstats.net/start.htm, accessed on 15 November 2021) devoted to the evaluation of the association of SNPs with diseases in the presence of other biological, genetic, or clinical variances.

## 3. Results

In our study we analyzed two populations of ESRD patients and controls, assessed in relation to demographic and clinical characteristics. As reported above, SNPs were chosen in relation to their possible involvement with renal disease and concomitant pathologies. Table 3 shows the allele and genotype frequencies in the distribution of single nucleotide polymorphisms, between subjects with chronic renal failure on dialysis therapy and controls.

The statistical analysis of the raw data did indicate that the *IL6 rs180795* and *MIF rs755622* SNPs crude frequencies of the patient group were significantly different with respect to the control group in particular *rs180795* C allele frequency, associated to reduced production of IL-6 is significantly reduced in ESRD patients whereas *rs755622* G positive genotypes is increased.

We then proceeded to apply logistic regression models, using codominant, dominant, and recessive models (chosen on the basis of BIC and AIC criteria) of inheritance and adjusting the results for biological variables (age, sex) and concomitant comorbidities (hypertension, diabetes, hypercholesterolemia).

The best results of multivariate analysis, applied to each of the nine selected SNPs (Table 4), allowed us to confirm that C positive genotypes of *MIF rs755622,* (dominant model) is an independent risk factor for ESDR patients (independent of age, gender, and associated pathologies). In addition, a rough significant influence of both *TNFA rs1800629* homozygous genotypes have been detected (complete model analyses for each SNP are reported in Supplementary Tables S1–S9).

To evaluate the possible interactions between SNPs and biological (age and gender) and clinical variables (hypertension, diabetes, and hypercholesterolemia), genotype frequencies of ESRD patients and controls were stratified and compared according to the different variables. Complete results of these analyses are reported in Supplementary Tables S10–14.

Stratifying results according to age, *MIF rs755622 C* positive genotype frequencies are increased both in <59 and ≥59-year-old patients than in controls (Table 5). No other SNP has been shown to play a role in ESRD predisposition in association with age cut-off (Supplementary Table S10).

Analyses of data according to gender allowed us to observe that ESRD women shoved a significantly reduced frequency of genotypes bearing the *IL6 rs180795 C* allele. No differences were observed for men (Table 6).

No other SNP has been shown to play a role in ESRD predisposition in association with gender (Supplementary Table S11).

Next, we evaluated the genotype frequency distribution of the nine SNPs, in relation to the presence of comorbidities as covariances. The power calculation was analyzed for the statistical analyses performed, when data were stratified for hypertension diabetes and hypercholesterolemia power of calculation was weak, so the statistical significance of these data was not considered to be relevant. No differences in frequencies of the SNP genotypes in ESRD patients affected by hypertension with respect to no hypertensive ESDR and hypertensive control groups (see Supplementary Table S12).

On the other hand, 15 of the 19 ESDR Diabetic patients were positive for homozygous GG genotype of *MIF rs755622* with a significant increase of frequency with respect to non-diabetic patients (Supplementary Table S13). When genotype frequencies were analyzed according to the presence of the Hypercholesterolemia of 22 ESRD-affected patients, 9 were positive for C allele bearing genotype of *MIF rs755622* with an increase of genotype frequencies with respect to hypercholesterolemic controls (Supplementary Table S14).

All in all, our results indicate that *MIF rs755622* might influence the susceptibility to ESDR both as an independent risk factor and in presence of comorbidities.

## 4. Discussion

The last stage of chronic renal failure (ESRD) is the result of the progressive reduction of renal function and the onset of a progressively more severe chronic renal failure whose evolution is strongly influenced by the underlying disease, age, sex, and genetic background of the patients. If we consider the progressive aging trend of the population and the increase in the prevalence of the main diseases predisposing to chronic kidney failure (CKF) (diabetes, hypertension, and cardiovascular diseases), identifying predicting genetic markers that allow early identification of patients with an increased risk to develop severe CKF and reach the ESRD appears to be mandatory [52,53].

Analyses of alleles and genotypes crude frequencies of nine SNPs of genes codifying for key cytokine and receptors involved in kidney damage pathogenesis do not allow to identify associations with the *Klotho*, in spite of the above-mentioned data on the role of *rs577912* polymorphism as risk factors of mortality in dialyzed patients [13] and the role of *rs9536314* in the so-called longevity trait [17]. In a study performed in 2016, it was shown that the GG genotype is associated with reduced survival and poor prognosis in dialysis patients compared to the TT and TG genotypes [18] but we were unable to confirm this observation in our population. Similarly, *FGF23 rs7955866* and *IGF1 rs35767* were not found to be associated with ESRD. On the other hand, more recently, other *FGF23* SNPs have been associated with CKD [54]. So further studies are warranted on this point. Regarding IGF-1, this cytokine plays major a role in the main pathways in the progression of metabolic traits, such as progression of T2DM complications, as CKD, and the development of cardiovascular disease. In particular, *rs35767* is known to contribute to the development of diabetes in various populations [25].

Interleukin-6 (IL-6), another important proinflammatory cytokine has been shown to play a critical role in the development of atherosclerosis and atherosclerotic disease including kidney diseases [55,56], and it is well known that patients having CKD are at higher risk for accelerated atherosclerosis [33]. Our results show that frequencies of genotypes bearing the C allele of *IL6 rs1800795* are lower in patients than in controls allowing us to hypothesize that the presence of the C allele might be protective against progression to ESRD. This was not confirmed by adjusting the odds ratio for biological variances and clinical comorbidities (Table 4), whereas the protective association was confirmed stratifying data according to gender in women but not in men.

It has long been known that the *rs1800795G/C* variant located in the gene promoter *(−174G/C*) is associated with changes in transcriptional expression [57].

Even if IL-6 tracks with disability and age-related diseases, published data on the interaction among *rs1800795* and gender are not conclusive. Studies on aging frailty or longevity trait have demonstrated the possibility that genetically determined high production of IL-6 and women gender are risk factors for age-related disease [58,59]. In this view, the IL-6 low production associated with the *rs1800795* C allele might be protective against ESRD for CKD-affected women. On the other hand, the relatively small size of the patient sample does not allow to consider this observation conclusive.

In diabetes, the C allele is associated with a low risk of nephropathy [60,61]. On the other hand, the C allele was found to be associated with sepsis, high levels of circulating IL-6, and mortality increase in dialyzed subjects [62,63,64].

These apparently conflicting results are not easy to interpret. On the other hand, genetically determined low production of IL-6 might be a vantage in the contrasting progression of systemic diseases characterized by a chronic low rate of the inflammatory response [65], as CKD, but might be detrimental in acute inflammatory response characterizing sepsis or dialysis complications.

As reported in Table 4, after correction for confounding factors, a weak association of *TNFA rs1800629* genotypes has been detected. Cytokines of the TNF family and their receptor has been recently proposed as biomarkers of kidney disease. Actually, TNF signaling pathways play important roles in the progression of atherosclerotic and kidney disease [31,32,33] and in particular in the progression of diabetic nephropathy [66,67].

Studies on *TNFA* SNPs have suggested that *rs1800629*, associated with an increased *TNFA* gene transcription, might be a risk factor for multifactorial diseases [68], in particular, *rs1800629* might be a risk factor for CKD in particular in diabetic patients but data have been not conclusive [38,39]. Generally, TNF-α concentrations are found to be elevated in various cardiovascular conditions (e.g., advanced health failure, cardiomyopathy) and may also induce vascular inflammation, which may contribute to the pathogenesis of atherosclerosis [69].

Arterial hypertension is one of the main causes of kidney damage leading to end-stage renal disease (ESRD) [70,71], and cardiovascular disease is the leading cause of mortality in patients with renal disease [72].

In spite of this, our data do not allow us to identify the association of hypertension and SNP genotype studied as risk or protective factors in our group of ESRD patients.

Typing of *MIF rs1007888*, which was found associated with an increased risk of diabetes [48], does not indicate a role for this SNP in ESRD predisposition, whereas the C positive genotypes of *rs755622*, located at −173bp before the transcription initiation site of *MIF* gene and associated with a higher production of the cytokine [73], is more frequent in patients than in controls. This observation was also confirmed by adjusting the data for confounding factors. In addition, C positive genotypes of *MIF rs755622* seem to be associated with an increased risk to develop an ESRD both in patients ≥59 and <59 years old.

Aging is per se a risk factor for kidney diseases and in particular for CKD. However, it is well known that glomerular filtration rate (eGFR) declines with aging and this might impinge on the risk of CKD evolution to terminal stage and mortality. As recently reported by Delanaye et al. [74], for people older than 65 years (reference eGFR 75–89 mL/min per 1.73 m^2^), the risk was relevant only when eGFR had fallen below 45 mL/min. In this view, the possibility to refine the analyses combining age and significant SNP markers could be useful for the best management of CKD prevention and therapy. However, our data indicate that the *MIF rs755622* C allele is an age and gender independent risk factor for ESRD progression

Our results are in good agreement with data of other groups indicating that high production of MIF is correlated with an increased susceptibility for a large number of inflammatory and metabolic diseases including chronic kidney failure [75,76,77,78,79]. However, in renal pathologies, MIF seems to act according to a model of antagonistic pleiotropism, in fact, in different clinical situations and in different stages of renal pathology it can exert both harmful and protective effects [47] which can be likened to the story of Dr. Jekyll and Mr. Hyde. Indeed, MIF can be considered a potential biomarker of kidney damage. Elevated urinary and plasma MIF levels correlate with acute renal failure [80], chronic kidney disease [77], and the polycystic kidney [81].

On the other hand, in chronic kidney diseases (CKD), MIF limits the pro-fibrotic and proinflammatory activation of tubular cells by counteracting the arrest of the cell cycle and promoting its regeneration; In addition, in acute kidney damage (AKI), MIF also counteracts the apoptosis of tubular cells with a protective effect [43,82,83]. So it is possible that the net effect of the presence of *rs755622* on CKD predisposition and ESRD progression might be influenced by individual biological factors such as aging [84] or the presence of comorbidities. As it is well known, the presence of metabolic inflammation-based diseases, in particular diabetes and atherosclerosis, constitute a high-risk factor for CKD and progression towards ESRD [85].

As it is well known, diabetes is one of the major comorbidities associated with CKD. CKD can be a consequence of the alteration of the glucose status or we can have a kidney disease on which type II diabetes is superimposed. In diabetic nephropathy, the endothelial involvement and local induction of cytokines and chemokines induce immune cell recruitment accompanying and promoting tissue remodeling and nephron atrophy [86]. Moreover, CKD patients often have both quantitative and qualitative alterations in the lipid metabolism of lipoproteins. These lipid changes are present in all stages of the disease but differ in part on the basis of the deficit of renal function, the presence of a replacement treatment (dialysis and transplant), and the extent of proteinuria. The most frequently observed modification is the constant reduction in HDL, and hypercholesterolemia is a risk factor for chronic kidney disease [87,88].

The analysis of the association between the polymorphisms we typed and the risk of dialysis in subjects suffering from hypercholesterolemia or diabetes do not allow us to identify associations with polymorphisms known to be associated with changes in lipid and carbohydrate metabolism as *Klotho rs564481* [15,16], whereas *MIF rs755622* genetic polymorphism might play a role in ESRD progression in subjects affected by these pathologies. However, our data were obtained analyzing a relatively low number of subjects bearing diabetes or hypercholesterolemia, so a definitive conclusion cannot be drawn, even if some literature reports seem to support this view [78,89,90].

## 5. Conclusions

In conclusion, CKD might be considered a paradigm of the multifactorial diseases in which preexisting pathological conditions and genetic susceptibility factors might interplay with each other, resulting in different trajectories of damage evolution to the final stage as ESRD. In this view, we have analyzed the role of some genetic variants of key cytokines, mediators, and receptors involved in the complex scenario of kidney chronic failure and their interaction with comorbidities. Our data indicate that some polymorphisms, *IL6* *rs1800795* and *MIF rs755622*, involved in the regulation of both renal function and inflammatory response can influence the evolution of chronic kidney disease. In particular, *IL6* *rs1800795G* genotype and *MIF rs755622C* alleles associated with increased production of the respective cytokines inducing increased inflammatory damages of kidney parenchyma might be useful as a marker for evaluation of ESRD evolution risk.

In addition, it is hypothesized that the modulation of the activities of these and other genes can also be considered therapeutic targets for innovative therapies. In this light, further studies will certainly be needed to investigate the role and interaction of these SNPs, in particular *MIF rs755622*, in the progression of end-stage renal disease.

## Figures and Tables

**Table 1:** Demographic and clinical characteristics of 93 patients with end-stage renal disease (ESRD) and 213 control subjects.

Demographic and Clinical Characteristics	ESRD		Controls		<59-Year-Old Controls		≥59-Year-Old Controls		*p* *
	N.	%	N.	%	N.	%	N.	%	
Age, mean ± SD	69.01 ± 13.02		55.97 ± 10.99		43.75 ± 5.46		66.32 ± 5.24		0.1118
>59 years old	71	76.3	67	31.45	-	-	67	31.45	-
Women	42	45.16	94	44.13	64	43.83	30	44.78	1.000
	Pathologies in ESRD and Controls								
Hypertension	32	34.41	17	8.01	-	-	17	8.01	<0.0001
Type II Diabetes	19	20.43	8	3.76	-	-	8	3.76	<0.0001
Hypercholesterolemia	13	13.98	21	9.85	-	-	21	9.85	0.3242

* analyses of significant differences between ESRD patients and ≥59-year-old controls.

**Table 2:** Typed SNPs.

Gene	SNP	Major Allele	Minor Allele	Chr Location
*Klotho*	*rs577912*	C	A	13:33036014
	*rs564481*	C	T	13: 33060846
	*rs9536314*	T	G	13:33054001
*FGF23*	*rs7955866*	G	A	12:4370383
*IGF1*	*rs35767*	C	T	12:102481791
*TNFA*	*rs1800629*	G	A	6:31575254
*IL6*	*rs1800795*	G	C	7:22727026
*MIF*	*rs755622*	G	C	22:23894205
	*rs1007888*	T	C	22:23898914

**Table 3:** SNP allelic and genotypic frequencies of 93 dialyzed patients (ESRD) and 213 control subjects.

GENE	SNP	Alleles/ Genotypes	Controls		ESRD		OR (95% CI)	^1^*p*-Value
			Nr	Freq.	Nr	Freq.		
***Klotho***	***rs577912***	C	360	0.85	162	0.87	1.24 (0.75–2.05)	0.458
		A	66	0.15	24	0.13		
		CC	150	0.7	71	0.76		
		CA	60	0.28	20	0.22		0.442
		AA	3	0.01	2	0.02		
	***rs564481***	C	262	0.62	113	0.61	0.97 (0.68–1.38)	0.857
		T	164	0.38	73	0.39		
		CC	82	0.38	34	0.37		
		CT	98	0.46	45	0.48		0.927
		TT	33	0.15	14	0.15		
	***rs9536314***	T	355	0.83	154	0.83	0.96 (0.61–1.52)	0.907
		G	71	0.17	32	0.17		
		TT	153	0.72	63	0.68		
		TG	49	0.23	28	0.3		0.242
		GG	11	0.05	2	0.02		
***FGF23***	***rs7955866***	G	380	0.89	168	0.9	1.13 (0.64–2.01)	0.774
		A	46	0.11	18	0.1		
		GG	175	0.82	77	0.83		
		GA	30	0.14	14	0.15		0.757
		AA	8	0.04	2	0.02		
***IGF1***	***rs35767***	C	348	0.82	150	0.81	0.93 (0.60–1.45)	0.822
		T	78	0.18	36	0.19		
		CC	146	0.69	64	0.69		
		CT	56	0.26	22	0.24		0.673
		TT	11	0.05	7	0.08		
***TNFA***	***rs1800629***	G	388	0.91	176	0.95	1.72 (0.84–3.54)	0.145
		A	38	0.09	10	0.05		
		GG	178	0.84	85	0.91		
		GA	32	0.15	6	0.06		0.105
		AA	3	0.01	2	0.02		
***IL6***	***rs1800795***	G	311	0.73	152	0.82	0.61 (0.39–0.93)	0.024
		C	115	0.27	34	0.18		
		GG	118	0.55	65	0.70		
		GC	75	0.35	22	0.24		0.059
		CC	20	0.09	6	0.06		
***MIF***	***rs755622***	G	301	0.71	108	0.58	1.83 (1.12–2.99)	0.0028
		C	125	0.29	78	0.42		
		GG	128	0.60	42	0.45		
		GC	45	0.21	24	0.26		0.042
		CC	40	0.19	27	0.29		
	***rs1007888***	T	231	0.54	107	0.58	1.14 (0.81–1.62)	0.480
		C	195	0.46	79	0.42		
		TT	70	0.33	34	0.37		
		TC	91	0.43	39	0.42		0.778
		CC	52	0.24	20	0.22		

^1^ Evaluation of SNP alleles and genotypes, significant differences between ESRD and controls (crude analyses) was performed using the chi-square test or Fisher’s exact test applied to 2 × 2 or 3 × 2 contingency tables.

**Table 4:** Evaluation of SNP associations with ESRD (adjusted by age, gender, hypertension, diabetes, and hypercholesterolemia).

Gene	SNP	Genetic Models	OR (95% CI)	*p*-Value
	*rs577912*	Dominant	0.61 (0.32–1.20)	0.15
*Klotho*	*rs564481*	Dominant	1.09 (0.60–1.98)	0.77
	*rs9536314*	Recessive	0.25 (0.05–1.29)	0.067
*FGF23*	*rs7955866*	Recessive	0.90 (0.16–4.98)	0.9
*IGF1*	*rs35767*	Recessive	1.27 (0.39–4.12)	0.69
*TNFA*	*rs1800629*	Overdominant	**0.35 (0.12** **–** **1.06)**	**0.046**
*IL6*	*rs1800795*	Dominant	0.76 (0.42–1.39)	0.37
*MIF*	*rs755622*	Dominant	**3.37 (1.80** **–** **6.30)**	**<0.0001**
	*rs1007888*	Dominant	0.87 (0.48–1.58)	0.65

Evaluation of SNP associations with ESRD was performed using the best genetic transmission model of minor allele according to the lowest values of Bayesian and Akaike information criteria. Significant OR, and 95% CI are reported in bold.

**Table 5:** Significant SNP genotype frequencies (gen. freq.) in end-stage renal disease patients (ESRD) and controls (CTRL) stratified according to 59 years age cut off (adjusted by gender, diabetes, hypertension, and hypercholesterolemia).

Genes and SNP Alleles		Age < 59				Age ≥ 59			
		CTRL	ESRD	OR (95% CI)	*p*-Value	CTRL	ESRD	OR (95% CI)	*p*-Value
		N (Gen. Freq.)				N (Gen. Freq.)			
*MIF* *rs755622*	G/G	82 (0.56)	6 (0.28)	0.29 (0.11–0.79)	0.021	46 (0.69)	36 (0.51)	0.47 (0.23–0.94)	0.038
	C/G	29 (0.20)	8 (0.36)	3.95 (1.19–13.17)	0.013	16 (0.24)	16 (0.22)	0.93 (0.73–2.44)	1.00
	C/C	35 (0.24)	8 (0.36)	1.81 (0.70–4.68)	0.292	5 (0.07)	19 (0.27)	4.56 (1.69–12.97)	0.003
	C/*	64 (0.44)	16 (0.72)	3.30 (1.40–10.89)	0.021	21 (0.31)	35 (0.49)	2.13 (1.06–4.27)	0.038

**Table 6:** Significant SNP genotype frequencies (gen. freq.) in end-stage renal disease patients (ESRD) and controls (CTRL) stratified according to Gender (adjusted by 59 years of age cut-off, diabetes, hypertension, and hypercholesterolemia).

Genes and SNP Alleles		FEMALE				MALE			
		CTRL	ESRD	OR (95% CI)	*p*-Value	CTRL	ESRD	OR (95% CI)	*p*-Value
		N (Gen. Freq.)				N (Gen. Freq.)			
*IL6* *rs1800795*	G/G	50 (0.53)	31 (0.74)	2.48 (1.12–5.51)	0.036	68 (0.57)	34 (0.67)	1.50 (0.76–2.98)	0.306
	C/G	34 (0.36)	9 (0.21)	0.63 (0.23–1.69)	0.111	41 (0.35)	13 (0.25)	0.65 (0.32–1.38)	0.284
	C/C	10 (0.11)	2 (0.05)	0.49 (0.09–2.67)	0.342	10 (0.08)	4 (0.08)	0.92 (0.27–3.13)	1.00
	C/*	44 (0.47)	11 (0.26)	0.40 (0.18–0.89)	0.036	51 (0.43)	17 (0.33)	0.67 (0.34–1.32)	0.306

## Data Availability

All data generated or analyzed during this study are stored in electronic archives that can be supplied on request.

## Supplementary Materials

The following supporting information can be downloaded at: https://www.mdpi.com/article/10.3390/genes13020226/s1, Table S1. Evaluation of *Klotho rs577912* associations with ESRD (adjusted by age, gender, hypertension, diabetes, and hypercholesterolemia) using different genetic transmission models of minor alleles; Table S2. Evaluation of *Klotho rs564481* associations with ESRD (adjusted by age, gender, hypertension, diabetes, and hypercholesterolemia) using different genetic transmission models of minor alleles; Table S3. Evaluation of *Klotho rs9536314* associations with ESRD (adjusted by age, gender, hypertension, diabetes, and hypercholesterolemia) using different genetic transmission models of minor alleles; Table S4. Evaluation of *FGF23 rs7955866* associations with ESRD (adjusted by age, gender, hypertension, diabetes, and hypercholesterolemia) using different genetic transmission models of minor alleles; Table S5. Evaluation of *IGF1 rs35767* associations with ESRD (adjusted by age, gender, hypertension, diabetes, and hypercholesterolemia) using different genetic transmission models of minor alleles; Table S6. Evaluation of *TNFA rs1800629* associations with ESRD (adjusted by age, gender, hypertension, diabetes, and hypercholesterolemia) using different genetic transmission models of minor alleles; Table S7. Evaluation of *IL6 rs1800795* associations with ESRD (adjusted by age, gender, hypertension, diabetes, and hypercholesterolemia) using different genetic transmission models of minor alleles; Table S8. Evaluation of *MIF rs755622* associations with ESRD (adjusted by age, gender, hypertension, diabetes, and hypercholesterolemia) using different genetic transmission models of minor alleles; Table S9. Evaluation of *MIF rs1007888* associations with ESRD (adjusted by age, gender, hypertension, diabetes, and hypercholesterolemia) using different genetic transmission models of minor alleles; Table S10: SNP frequencies in end-stage renal disease patients (ESRD) and controls (CTRL) stratified according to 59 years age cut off (adjusted by gender, diabetes, hypertension, and hypercholesterolemia); Table S11: SNP frequencies in end-stage renal disease patients (ESRD) and controls (CTRL) stratified according to gender (adjusted by 59 years age cut off, diabetes, hypertension, and hypercholesterolemia); Table S12: SNP frequencies in end-stage renal disease patients affected by hypertension (Hy ESRD) compared to ESRD patients with normal blood pressure (No Hy ESRD) and hypertensive controls (HyCTRL) (adjusted by 59 years age cut off, gender, diabetes, and hypercholesterolemia); Table S13: SNP frequencies in end-stage renal disease patients affected by diabetes mellitus (DM ESRD) compared to ESRD patients without diabetes (No T2DM ESRD) and diabetic controls (DM CTRL) (adjusted by 59 years age cut off, gender, hypertension, and hypercholesterolemia); Table S14: SNP frequencies in end-stage renal disease patients affected by hypercholesterolemia (HC ESRD) compared to ESRD patients without hypercholesterolemia (No HC ESRD) and hypercholesterolemic controls (HC CTRL) (adjusted by 59 years age cut off, gender, hypertension, and diabetes).
