# Supplementary material for: MIF rs755622 and IL6 rs1800795 Are Implied in Genetic Susceptibility to End-Stage Renal Disease (ESRD)
Source: Genes (Basel). 2022 Jan 25;13(2):226. doi: 10.3390/genes13020226 (PMC8872268; doi:10.3390/genes13020226)
Supplement: Supplementary file 1 [file genes-13-00226-s001.zip › genes-1555606-SI.pdf]

Table S1. Evaluation of *Klotho rs577912* associations with ESRD (adjusted by age, gender, hypertension, diabetes and hypercholesterolemia) using different genetic transmission model of minor allele.

Model	Genotype	Controls N (%)	ESRD N (%)	OR (95% CI)	P-value	AIC	BIC
Codominant	C/C	150 (70.4%)	71 (76.3%)	1.00	0.16	305.5	335.3
	A/C	60 (28.2%)	20 (21.5%)	0.55 (0.28-1.11)			
	A/A	3 (1.4%)	2 (2.1%)	2.09 (0.29-15.01)			
Dominant	C/C	150 (70.4%)	71 (76.3%)	1.00	0.15	304	330.1
	A/C-A/A	63 (29.6%)	22 (23.7%)	0.61 (0.32-1.20)			
Recessive	C/C-A/C	210 (98.6%)	91 (97.8%)	1.00	0.4	306.4	332.5
	A/A	3 (1.4%)	2 (2.1%)	2.37 (0.33-16.82)			
Overdominant	C/C-A/A	153 (71.8%)	73 (78.5%)	1.00	0.078	305	331.1
	A/C	60 (28.2%)	20 (21.5%)	0.54 (0.27-1.09)			

Table S2. Evaluation of *Klotho rs564481* associations with ESRD (adjusted by age, gender, hypertension, diabetes and hypercholesterolemia) using different genetic transmission model of minor allele.

Model	Genotype	Controls N (%)	ESRD N (%)	OR (95% CI)	P-value	AIC	BIC
Codominant	C/C	83 (39%)	34 (36.6%)	1.00	0.87	308.9	338.7
	C/T	97 (45.5%)	45 (48.4%)	1.05 (0.56-1.98)			
	T/T	33 (15.5%)	14 (15.1%)	1.26 (0.53-2.97)			
Dominant	C/C	83 (39%)	34 (36.6%)	1.00	0.77	306.9	333.0
	C/T-T/T	130 (61%)	59 (63.4%)	1.09 (0.60-1.98)			
Recessive	C/C-C/T	180 (84.5%)	79 (85%)	1.00	0.62	307.0	333.1
	T/T	33 (15.5%)	14 (15.1%)	1.22 (0.56-2.67)			
Overdominant	C/C-T/T	116 (54.5%)	48 (51.6%)	1.00	0.96	307.1	333.2
	C/T	97 (45.5%)	45 (48.4%)	0.99 (0.56-1.75)			

Table S3. Evaluation of *Klotho rs9536314* associations with ESRD (adjusted by age, gender, hypertension, diabetes and hypercholesterolemia) using different genetic transmission model of minor allele.

Model	Genotype	Controls N (%)	ESRD N (%)	OR (95% CI)	P-value	AIC	BIC
Codominant	T/T	153 (71.8%)	63 (67.7%)	1.00	0.17	305.5	335.3
	G/T	49 (23%)	28 (30.1%)	1.18 (0.61-2.26)			
	G/G	11 (5.2%)	2 (2.1%)	0.27 (0.05-1.37)			
Dominant	T/T	153 (71.8%)	63 (67.7%)	1.00	0.9	307.1	333.2
	G/T-G/G	60 (28.2%)	30 (32.3%)	0.96 (0.52-1.79)			
Recessive	T/T-G/T	202 (94.8%)	91 (97.8%)	1.00	0.067	303.8	329.8
	G/G	11 (5.2%)	2 (2.1%)	0.25 (0.05-1.29)			
Overdominant	T/T-G/G	164 (77%)	65 (69.9%)	1.00	0.45	306.6	332.6
	G/T	49 (23%)	28 (30.1%)	1.28 (0.67-2.44)			

Table S4. Evaluation of *FGF-23 rs7955866* associations with ESRD (adjusted by age, gender, hypertension, diabetes and hypercholesterolemia) using different genetic transmission model of minor allele.

Model	Genotype	Controls N (%)	ESRD N (%)	OR (95% CI)	P-value	AIC	BIC
Codominant	G/G	175 (82.2%)	77 (82.8%)	1.00	0.96	309.1	338.9
	A/G	30 (14.1%)	14 (15.1%)	1.12 (0.48-2.63)			
	A/A	8 (3.8%)	2 (2.1%)	0.91 (0.16-5.08)			
Dominant	G/G	175 (82.2%)	77 (82.8%)	1.00	0.85	307.1	333.2
	A/G-A/A	38 (17.8%)	16 (17.2%)	1.08 (0.49-2.36)			
Recessive	G/G-A/G	205 (96.2%)	91 (97.8%)	1.00	0.9	307.0	333.1
	A/A	8 (3.8%)	2 (2.1%)	0.90 (0.16-4.98)			
Overdominant	G/G-A/A	183 (85.9%)	79 (85%)	1.00	0.79	307.1	333.2
	A/G	30 (14.1%)	14 (15.1%)	1.12 (0.48-2.63)			

Table S5. Evaluation of *IGF-1 rs35767* associations with ESRD (adjusted by age, gender, hypertension, diabetes and hypercholesterolemia) using different genetic transmission model of minor allele.

Model	Genotype	Controls N (%)	ESRD N (%)	OR (95% CI)	P-value	AIC	BIC
Codominant	C/C	146 (68.5%)	64 (68.8%)	1.00	0.9	308.9	338.7
	C/T	56 (26.3%)	22 (23.7%)	0.92 (0.47-1.81)			
	T/T	11 (5.2%)	7 (7.5%)	1.24 (0.38-4.09)			
Dominant	C/C	146 (68.5%)	64 (68.8%)	1.00	0.94	307.1	333.2
	C/T-T/T	67 (31.5%)	29 (31.2%)	0.98 (0.53-1.82)			
Recessive	C/C-C/T	202 (94.8%)	86 (92.5%)	1.00	0.69	307.0	333.0
	T/T	11 (5.2%)	7 (7.5%)	1.27 (0.39-4.12)			
Overdominant	C/C-T/T	157 (73.7%)	71 (76.3%)	1.00	0.77	307.1	333.1
	C/T	56 (26.3%)	22 (23.7%)	0.90 (0.46-1.76)			

Table S6. Evaluation of *TNF rs1800629* associations with ESRD (adjusted by age, gender, hypertension, diabetes and hypercholesterolemia) using different genetic transmission model of minor allele.

Model	Genotype	Controls N (%)	ESRD N (%)	OR (95% CI)	p-value	AIC	BIC
Codominant	G/G	178 (83.6%)	85 (91.4%)	1.00	0.12	304.8	334.6
	A/G	32 (15%)	6 (6.5%)	0.36 (0.12-1.07)			
	A/A	3 (1.4%)	2 (2.1%)	1.90 (0.21-17.58)			
Dominant	G/G	178 (83.6%)	85 (91.4%)	1.00	0.11	304.6	330.6
	A/G-A/A	35 (16.4%)	8 (8.6%)	0.47 (0.18-1.24)			
Recessive	G/G-A/G	210 (98.6%)	91 (97.8%)	1.00	0.52	306.7	332.8
	A/A	3 (1.4%)	2 (2.1%)	2.10 (0.23-19.42)			
Overdominant	G/G-A/A	181 (85%)	87 (93.5%)	1.00	0.046	303.2	329.2
	A/G	32 (15%)	6 (6.5%)	0.35 (0.12-1.06)			

Table S7. Evaluation of *IL-6 rs1800795* associations with ESRD (adjusted by age, gender, hypertension, diabetes and hypercholesterolemia) using different genetic transmission model of minor allele.

Model	Genotype	Controls N (%)	ESRD N (%)	OR (95% CI)	P-value	AIC	BIC
Codominant	G/G	118 (55.4%)	65 (69.9%)	1.00	0.6	308.1	337.9
	C/G	75 (35.2%)	22 (23.7%)	0.81 (0.42-1.56)			
	C/C	20 (9.4%)	6 (6.5%)	0.61 (0.20-1.81)			
Dominant	G/G	118 (55.4%)	65 (69.9%)	1.00	0.37	306.3	332.4
	C/G-C/C	95 (44.6%)	28 (30.1%)	0.76 (0.42-1.39)			
Recessive	G/G-C/G	193 (90.6%)	87 (93.5%)	1.00	0.42	306.5	332.6
	C/C	20 (9.4%)	6 (6.5%)	0.65 (0.22-1.91)			
Overdominant	G/G-C/C	138 (64.8%)	71 (76.3%)	1.00	0.65	306.9	333
	C/G	75 (35.2%)	22 (23.7%)	0.86 (0.45-1.64)			

Table S8. Evaluation of *MIF rs755622* associations with ESRD (adjusted by age, gender, hypertension, diabetes and hypercholesterolemia) using different genetic transmission model of minor allele.

Model	Genotype	Controls N (%)	ESRD N (%)	OR (95% CI)	P-value	AIC	BIC
Codominant	G/G	128 (60.1%)	42 (45.2%)	1.00	1e-04	291	320.8
	C/G	45 (21.1%)	24 (25.8%)	2.48 (1.18-5.21)			
	C/C	40 (18.8%)	27 (29%)	4.81 (2.22-10.42)			
Dominant	G/G	128 (60.1%)	42 (45.2%)	1.00	1e-04	290.8	317.5
	C/G-C/C	85 (39.9%)	51 (54.8%)	3.37 (1.80-6.30)			
Recessive	G/G-C/G	173 (81.2%)	66 (71%)	1.00	4e-04	294.7	320.8
	C/C	40 (18.8%)	27 (29%)	3.52 (1.73-7.17)			
Overdominant	G/G-C/C	168 (78.9%)	69 (74.2%)	1.00	0.24	305.8	331.8
	C/G	45 (21.1%)	24 (25.8%)	1.49 (0.77-2.90)			

Table S9. Evaluation of *MIF rs1007888* associations with ESRD (adjusted by age, gender, hypertension, diabetes and hypercholesterolemia) using different genetic transmission model of minor allele.

Model	Genotype	Controls N (%)	ESRD N (%)	OR (95% CI)	P-value	AIC	BIC
Codominant	T/T	70 (32.9%)	34 (36.6%)	1.00	0.81	308.7	338.5
	C/T	91 (42.7%)	39 (41.9%)	0.92 (0.48-1.77)			
	C/C	52 (24.4%)	20 (21.5%)	0.77 (0.35-1.69)			
Dominant	T/T	70 (32.9%)	34 (36.6%)	1.00	0.65	306.5	332.0
	C/T-C/C	143 (67.1%)	59 (63.4%)	0.87 (0.48-1.58)			
Recessive	T/T-C/T	161 (75.6%)	73 (78.5%)	1.00	0.54	306.8	332.8
	C/C	52 (24.4%)	20 (21.5%)	0.81 (0.40-1.62)			
Overdominant	T/T-C/C	122 (57.3%)	54 (58.1%)	1.00	0.94	307.1	333.2
	C/T	91 (42.7%)	39 (41.9%)	1.02 (0.58-1.82)			

Table S10. SNP frequencies in End Stage Renal Disease Patients (ESRD) and Controls (CTRL) stratified according to 59 years Age cut off (adjusted by Gender, Diabetes, Hypertension and Hypercholesterolemia)

**Genes and SNP alleles**		**Age <59**				**Age ≥59**			
		**CTRL**	**ESRD**	**OR (95% CI)**	**p-value**	**CTRL**	**ESRD**	**OR (95% CI)**	**p-value:**
KLOTHO	**C/C**	104	19	2.56 (0.72-9.10)	0.196	46	52	1.25 (0.59-2.61)	0.578
rs577912	**A/C**	40	2	0.21 (0.04-1.08)	0.109	20	18	0.76 (0.33-1.72)	0.689
	**A/A**	2	1	2.77 (0.24-32.66)	0.345	1	1	1.42 (0.08-24.29)	1.00
	A/*	42	3	0.39 (0.11-1.39)	0.196	21	19	0.80 (0.38-1.67)	0.578
rs564481	**C/C**	60	4	3.14 (1.01-9.74)	0.057	22	30	1.49 (0.75-2.99)	0.293
	**C/T**	63	10	2.33 (0.68-8.00)	0.100	35	35	0.73 (0.34-1.59)	0.862
	**T/T**	23	8	0.33 (0.12-0.87)	0.035	10	6	0.44 (0.13-1.58)	0.292
	T/*	86	18	0.32 (0.103-0.99)	0.057	45	41	0.67 (0.33-1.34)	0.293
rs953614	**T/T**	109	19	0.46 (0.13-1.66)	0.351	44	44	0.85 (0.42-1.71)	0.724
	**G/T**	33	3	1.85 (0.52-6.64)	0.416	16	25	1.66 (0.73-3.74)	0.192
	**G/G**	4	0	---	---	7	2	0.34 (0.06-1.86)	0.091
	G/*	37	3	2.15 (0.61-7.68	0.351	23	27	1.17 (0.59-2.35)	0.724
FGF23	**G/G**	116	18	0.86 (0.27-2.73)	1.00	59	59	0.67 (0.25-1.74)	0.473
rs7955866	**A/G**	24	3	0.79 (0.20-3.09)	1.00	6	11	1.49 (0.46-4.87)	0.304
	**A/A**	6	1	1.11 (0.11-10.73)	1.00	2	1	0.72 (0.06-8.92)	0.611
	A/*	30	4	1.16 (0.37-3.70)	1.00	8	12	1.50 (0.57-3.94)	0.473
IGF	**C/C**	102	14	1.42 (0.55-3.64)	0.617	44	50	1.24 (0.61-2.54)	0.587
rs35767	**C/T**	39	6	1.32 (0.45-3.83)	1.00	17	16	0.74 (0.31-1.74)	0.842
	**T/T**	5	2	3.09 (0.53-17.93)	0.229	6	5	0.74 (0.17-3.25)	0.759
	T/*	41	8	0.70 (0.27-1.80)	0.617	23	21	0.80 (0.39-1.64)	0.587
TNF	**G/G**	120	20	0.46 (0.10-2.09)	0.377	58	65	1.68 (0.56-5.01)	0.418
rs1800629	**A/G**	23	2	0.57 (0.12-2.66)	0.535	9	4	0.25 (0.06-1.09)	0.149
	**A/A**	3	0	---	---	0	2	---	---
	A/*	26	2	2.17 (0.48-9.85)	0.377	9	6	0.59 (0.19-1.77)	0.418
IL6	**G/G**	75	14	0.60 ()0.24-1.53)	0.361	43	51	1.42 (0.69-2.92)	0.365
rs1800795	**C/G**	59	6	4.72 (0.67-5.05)	0.254	16	16	1.09 (0.38-2.11)	1.00
	**C/C**	12	2	0.92 (0.18-4.84)	1.00	8	4	0.48 (0.12-1.87)	0.312
	C/*	71	8	1.66 (0.65-4.18)	0.361	24	20	0.70 (0.34-1.44)	0.365
MIF	**G/G**	82	6	0.29 (0.11-0.79)	0.021	46	36	0.47 (0.23-0.94)	0.038
rs755622	**C/G**	29	8	3.95 (1.19-13.17)	0.013	16	16	0.93 (0.73-2.44)	1.00
	**C/C**	35	8	1.81 (0.70-4.68)	0.292	5	19	4.56 (1.69-12. 97)	0.003
	C/*	64	16	3.30 (1.40-10.89)	0.021	21	35	2.13 (1.06-4.27)	0.038
rs1007888	**T/T**	45	7	1.05 (0.39-2.74)	1.00	25	27	1.03 (0.52-2.05)	1.00
	**C/T**	64	9	0.83 (0.28-2.44)	0.822	27	30	1.09 (0.44-2.22)	0.863
	**C/C**	37	6	1.10 (0.40-3.03)	1.00	15	14	0.75 (0.27-2.05)	0.834
	C/*	101	15	0.95 (0.36-2.50)	1.00	42	44	0.97 (0.49-1.93)	1.00

Table S11. SNP frequencies in End Stage Renal Disease Patients (ESRD) and Controls (CTRL) stratified according to Gender (adjusted by 59 years Age cut off, Diabetes, Hypertension and Hypercholesterolemia)

Genes and SNP alleles		FEMALE				MALE			
		**CTRL**	**ESRD**	**OR (95% CI)**	**p-value**	**CTRL**	**ESRD**	**OR (95% CI)**	**p-value:**
KLOTHO	**C/C**	71	30	0.81 (0.36-1.83)	0.673	79	41	2.08 (0.94-4.57)	0.097
rs577912	**A/C**	21	10	1.09 (0.33-2.65)	1.00	39	10	0.50 (0.22-1.10)	0.098
	**A/A**	2	2	2.61 (0.53-16.3)	0.587	1	0	---	---
	**A/***	23	12	1.23 (0.54-2.80)	0.673	40	10	0.48 (0.22-1.06)	0.097
rs564481	**C/C**	33	15	1.03 (0.48-2.19)	1.00	49	19	0.85 (0.43-1.67)	0.733
	**C/T**	45	21	1.02 (0.30-2.00)	0.854	53	24	1.33 (0.57-2.14)	0.867
	**T/T**	16	6	0.82 (0.38-2.63)	0.804	17	8	1.17 (0.36-3.18)	0.999
	**T/***	61	27	0.97 (0.45-2.08)	1.00	70	32	1.18 (0.60-2.32)	0.733
rs953614	**T/T**	66	27	0.76 (035-1.65)	0.551	87	36	0.88 (0.43-1.82)	0.851
	**G/T**	24	15	1.24 (0.49-3.11)	0.305	25	13	1.23 (0.59-2.77)	0.549
	**G/G**	4	0	---	---	7	2	0.42 (0.17-3.37)	0.725
	**G/***	28	15	1.31 (0.61-2.83)	0.551	32	15	1.13 (0.55-2.34)	0.851
FGF23	**G/G**	77	35	1.10 (0.42-2.90)	1.00	98	42	1.00 (0.42-2.36)	1.00
rs7955866	**A/G**	14	6	0.52 (0.18-1.62)	0.235	16	8	1.19 (0.47-3.02)	0.810
	**A/A**	3	1	0.69 (0.07-5.77)	0.633	5	1	0.46 (0.05-3.99)	0.669
	**A/***	17	7	0.91 (0.34-2.38)	1.00	21	9	1.00 (0.42-2.36)	1.00
IGF	**C/C**	70	27	0.62 (0.28-1.35)	0.304	76	37	1.49 (0.73-3.07)	0.293
rs35767	**C/T**	21	13	1.58 (0.60-4.16)	0.393	35	9	0.56 (0.21-1.17)	0.128
	**T/T**	3	2	2.65 (0.27-10.1)	0.391	8	5	1.51 (0.46-4.85)	0.534
	**T/***	24	15	1.62 (0.74-3.54)	0.304	43	14	0.67 (0.33-1.37)	0.293
TNF	**G/G**	77	39	2.87 (0.79-10.4)	0.119	101	46	1.64 (0.57-4.69)	0.465
rs1800629	**A/G**	16	2	0.20 (0.03-1.24)	0.058	16	4	0.55 (0.17-1.72)	0.437
	**A/A**	1	1	2.32 (0.10-51.5)	1.00	2	1	1.17 (0.10-13.20)	1.00
	**A/***	17	3	0.35 (0.09-1.26)	0.119	18	5	0.61 (0.21-1.74)	0.465
IL6	**G/G**	50	31	2.48 (1.12-5.51)	0.036	68	34	1.50 (0.76-2.98)	0.306
rs1800795	**C/G**	34	9	0.63 (0.23-1.69)	0.111	41	13	0.65 (0.32-1.38)	0.284
	**C/C**	10	2	0.49 (0.09-2.67)	0.342	10	4	0.92 (0.27-3.13)	1.00
	**C/***	44	11	0.40 (0.18-0.89)	0.036	51	17	0.67 (0.34-1.32)	0.306
MIF	**G/G**	50	16	1.85 (0.88-3.88)	0.137	78	26	0.55 (0.28-1.06)	0.087
rs755622	**C/G**	18	10	2.87 (0.90-9.16)	0.647	27	14	1.28 (0.61-2.72)	0.559
	**C/C**	26	16	1.61 (0.74-3.47)	0.235	14	11	2.06 (0.86-4.92)	0.154
	**C/***	44	26	0.54 (0.26-1.14)	0.137	41	25	1.83 (0.94-3.56)	0.087
rs1007888	**T/T**	29	16	1.38 (0.64-2.95)	0.434	41	18	1.04 (0.52-2.06)	1.00
	**C/T**	40	18	1.84 (0.32-2.18)	1.00	51	21	0.99 (0.41-1.81)	0.867
	**C/C**	25	8	0.41 (0.12-1.39)	0.393	27	12	1.05 (0.48-2.28)	1.00
	**C/***	65	26	0.72 (0.34-1.55)	0.434	78	33	0.96 (0.48-1.92)	1.00

Table S12. SNP frequencies in End Stage Renal Disease Patients affected by Hypertension (Hy ESRD) compared to ESRD patients with Normal Blood Pressure (No Hy ESRD) and Hypertensive Controls (HyCTRL) (adjusted by 59 years Age cut off, Gender, Diabetes, and Hypercholesterolemia)

Genes and SNP alleles		Hy ESRD		No Hy ESRD		Hy CTRL		A		B	
		N	Freq.	N	Freq.	N	Freq.	OR (95% CI)	p value	OR (95% CI)	p value
KLOTHO	**C/C**	25	0.781	46	0.754	13	0.765	1.16 (0.42-3.2)	1.00	1.09 (0.27-4.45)	1.00
	**A/C**	7	0.219	13	0.213	4	0.235	1.03 (0.37- 2.92)	1.00	0.92 (0.22-3.65)	1.00
rs577912	**A/A**	0	---	2	0.033	0	---	---	---	---	---
	**A/***	7	0.219	15	0.246	4	0.235	0.86 (0.31-2.38)	1.00	0.92 (0.22-3.65)	1.00
	**C/C**	10	0.312	24	0.393	6	0.353	0.70 (0.28- 1.74)	0.502	0.83 0.24-2.89)	1.00
rs564481	**C/T**	16	0.500	29	0.476	7	0.412	1.10 (0.47-2.60)	0.831	1.42 (0.31-6.56)	0.764
	**T/T**	6	0.188	8	0.131	4	o.235	1.53 (0.48-4.8/)	0.546	0.76 (0.18-3.43)	0.721
	**T/***	22	0.688	37	0.607	11	0.647	1.43 (0.58- 3.53)	0.502	1.20 (0.34-4.16)	1.00
	**T/T**	22	0.688	41	0.672	12	0.706	1.07 (0.43- 2.69)	1.00	0.91 (0.25-3.31)	1.00
rs953614	**G/T**	10	0.312	18	0.295	3	0.176	1.09 (0.43-2.75)	1.00	1.97 (0.37-9.36)	0.339
	**G/G**	0	---	2	0.033	2	0.118	---	---	---	---
	**G/***	10	0.312	20	0.328	5	0.294	0.93 (0.37-2.34)	1.00	1.09 (0.30-3.94)	1.00
FGF23	**G/G**	24	0.750	53	0.869	14	0.824	0.45 (0.15-1.35)	0.161	0.64 (0.15-2.83)	0.725
	**A/G**	7	0.219	7	0.115	3	0.176	2.16 (0.68-6.82)	0.226	1.30 (0.24-5.72)	1.00
rs7955866	**A/A**	1	0.031	1	0.016	0	---	1.87 (0.11-31.00)	1.00	---	---
	**A/***	8	0.281	8	0.131	3	0.176	2.21 (0.74-6.58)	1.00	1.55 (0.35-6.84)	0.725
IGF	**C/C**	22	0.688	42	0.689	12	0.706	0.99 (0.39-2.51)	1.00	0.92 (0.25-3.31)	1.00
	**C/T**	6	0.188	16	0.262	5	0.294	0.65 (0.23-1.86)	0.456	0.56 (0.14-2.17)	0.479
rs35767	**T/T**	4	0.125	3	0.049	0	---	2.76 (0.58-13.19)	0.228	---	---
	**T/***	10	0.312	19	0.311	5	0.294	1.00 (0.40-2.53)	1.00	1.09 (0.30-3.95)	1.00
TNF	**G/G**	28	0.875	57	0.934	15	0.882	0.49 (0.11-2.11)	0.439	0.93 (0.15 (5.70)	1.00
	**A/G**	4	0.125	2	0.033	2	0.118	4.21 (0.73- 24.40)	0.176	1.08 (0.17-6.54)	1.00
rs1800629	**A/A**	0	---	2	0.033	0	---	---	---	---	---
	**A/***	4	0.125	4	0.066	2	0.118	2.04 (0.47-8.75)	0.439	1.08 (0.17-6.54)	1.00
IL6	**G/G**	23	0.719	42	0.689	10	0.588	1.16 (0.45-2.97)	0.816	1.79 (0.52-6.15)	0.523
	**C/G**	5	0.156	17	0.279	6	0.353	0.48 (0.16-1.45)	0.211	0.31 (0.07-1.43)	0.156
rs1800795	**C/C**	4	0.125	2	0.033	1	0.059	4.21 (0.73-24.40)	0.176	3.73 (0.29-22.53)	0.646
	**C/***	9	0.281	19	0.311	7	0.412	0.86 (0.34-2.22)	0.816	0.56 (0.16-1.92)	0.523
MIF	**G/G**	17	0.531	25	0.410	13	0.765	1.63 (0.69-3.86)	0.282	0.35 (0.09-1.30)	0.134
	**C/G**	9	0.281	15	0.246	4	0.235	1.20 (0.46-3.15)	0.804	1.32 (0.32-4.84)	1.00
rs755622	**C/C**	6	0.188	21	0.344	0	---	0.44 (0.16-1.23)	0.151	---	---
	**C/***	15	0.469	36	0.590	4	0.235	0.61 (0.26-1.45)	0.282	2.87 (0.77-10.72)	0.134
	**T/T**	10	0.313	24	0.393	3	0.176	0.70 (0.28-1.74)	0.502	2.12 (0.49-9.08)	0.339
rs1007888	**C/T**	13	0.406	26	0.426	7	0.412	0.92 (0.39-2.20)	1.00	0.99 (0.29-3.21)	1.00
	**C/C**	9	0.281	11	0.181	7	0.412	1.78 (0.65- 4.88)	0.295	0.57 (0.16-1.95)	0.523
	**C/***	22	0.687	37	0.607	14	0.824	1.43 (0.58-3.54)	0.502	0.47 (0.11-2.02)	0.339

A: Hy ESRD Vs No Hy ESRD

B: Hy ESRD Vs Hy CTRL

Table S13. SNP frequencies in End Stage Renal Disease Patients affected by Diabetes Mellitus (DM ESRD) compared to ESRD patients without diabetes (No T2DM ESRD) and Diabetic Controls (DM CTRL) (adjusted by 59 years Age cut off, Gender, hypertension, and hypercholesterolemia)

Genes and SNP alleles		DM ESRD		NoDM ESRD		DM CTRL		A		B	
		N	Freq.	N	Freq.	N	Freq.	OR (95% CI)	p value	OR (95% CI)	p value
KLOTHO	**C/C**	14	0.737	57	0.770	3	0.375	0.84 (0.26-2.65)	0.767	4.67 (0.77-5.00)	0.102
	**A/C**	5	0.263	15	0.203	5	0.625	1.41 (0.44-4.52)	0.546	0.17 (0.02-1.36)	0.102
rs577912	**A/A**	0	---	2	0.027	0	---	---	---	---	---
	**A/***	5	0.263	17	0.230	5	0.625	1.20 (0.38-3.80)	0.767	0.17 (0.02-1.36)	0.102
rs564481	**C/C**	9	0.474	25	0.338	4	0.500	1.76 (0.64-4.90)	0.296	1.11(0.21-5.80)	1.00
	**C/T**	7	0.368	38	0.514	3	0.375	0.55 (0.20-1.56)	0.310	0.88 (0.11-7.02)	1.00
	**T/T**	3	0.158	11	0.148	1	0.125	1.07 (0.27-4.31)	1.00	1.87 (0.14-14.26)	1.00
	**T/***	10	0.526	49	0.662	4	0.500	0.57 (0.20-1.58)	0.296	0.90 (0.17-4.70)	1.00
rs953614	**T/T**	16	0.842	47	0.635	6	0.750	3.06 (0.82-11.48)	0.104	1.78 (0.23-13.40)	0.616
	**G/T**	3	0.158	25	0.338	1	0.125	0.37 (0.10-1.38)	0.166	1.44 (0.13-14.23)	1.00
	**G/G**	0	---	2	0.027	1	0.125	---	---	---	---
	**G/***	3	0.158	27	0.365	2	0.250	0.33 (0.09-1.22)	0.104	0.56 (0.07-4.24)	0.616
FGF23	**G/G**	15	0.789	62	0.838	7	0.875	0.73 (0.20-2.57)	0.734	0.63 (0.05-8.65)	1.00
	**A/G**	4	0.211	10	0.135	1	0.125	1.71 (0.47-6.19)	0.474	1.87 (0.17-19.92)	1.00
rs7955866	**A/A**	0	---	2	0.027	0	---	---	---	---	---
	**A/***	4	0.211	12	0.162	1	0.125	1.38 (0.39-4.88)	0.734	1.87 (0.17-19.92)	1.00
IGF	**C/C**	11	0.579	53	0.716	7	0.875	0.54 (0.19-1.54)	0.275	0.19 (0.02-1.92)	0.201
	**C/T**	5	0.263	17	0.230	1	0.125	1.20 (0.38-3.80)	0.767	3.17 (0.24-24.71)	0.633
rs35767	**T/T**	3	0.158	4	0.054	0	---	3.28 (0.67-16.14)	0.148	---	---
	**T/***	8	0.421	21	0.284	1	0.125	1.83 (0.65-5.20)	0.275	5.09 (0.52-50.0)	0.201
TNF	**G/G**	15	0.789	70	0.946	8	1.00	0.214 (0.05-0.95)	0.052	---	---
	**A/G**	3	0.158	3	0.041	0	---	4.44 (0.82-24.05)	0.097	---	---
rs1800629	**A/A**	1	0.053	1	0.013	0	---	4.06 (0.24-68.04)	0.369	---	---
	**A/***	4	0.211	4	0.054	0	---	4.67 (1.05-20.80)	0.052	---	---
IL6	**G/G**	16	0.842	49	0.662	7	0.875	2.72 (0.72-10.22)	0.166	0.76 (0.07-8.66)	1.00
	**C/G**	2	0.105	20	0.270	1	0.125	0.32 (0.07-1.50)	0.224	1.11 (0.06-10.43)	1.00
rs1800795	**C/C**	1	0.053	5	0.068	0	----	0.77 (0.08-6.98)	1.00	---	---
	**C/***	3	0.158	25	0.338	1	0.125	0.37 (0.10-1.38)	0.166	1.31 (0.12-16.43)	1.00
MIF	**G/G**	15	0.789	27	0.365	7	0.875	6.53 (1.96-21.68)	0.0015	0.53 (0.05-5.72)	1.00
	**C/G**	1	0.053	23	0.311	1	0.125	0.12 (0.02-0.98)	0.021	0.44 (0.02-8.06)	1.00
rs755622	**C/C**	3	0.158	24	0.324	0	---	0.39 (0.10-1.47)	0.256	---	---
	**C/***	4	0.211	47	0.635	1	0.125	0.15 (0.05-0.51)	0.0015	1.87 (0.17-19.92)	1.00
rs1007888	**T/T**	7	0.368	27	0.365	3	0.375	1.02 (0.36-2.89)	1.00	0.97- (0.17-5.36)	1.00
	**C/T**	9	0.474	30	0.405	2	0.250	1.32 (0.48-3.64)	0.612	3.12 (0.36-16.95)	0.405
	**C/C**	3	0.158	17	0.230	3	0.375	0.63 (0.16-2.42)	0.755	0.48 (0.04-2.55)	0.319
	**C/***	12	0.632	47	0.635	5	0.625	0.98 (0.35-2.80)	1.00	1.02 (0.19-5.67)	1.00

A: DM ESRD Vs NoDM ESRD

B: DM ESRD Vs DM CTRL

Table S14. SNP frequencies in End Stage Renal Disease Patients affected by Hypercholesterolemia (HC ESRD) compared to ESRD patients without Hypercholesterolemia (No HC ESRD) and Hypercholesterolemic Controls (HC CTRL) (adjusted by 59 years Age cut off, Gender, hypertension and Diabetes)

Genes and SNP	alleles	HC ESRD		No HC ESRD		HC CTRL		A		B	
		N	Freq.	N	Freq.	N	Freq.	OR (95% CI)	p value	OR (95% CI)	p value
KLOTHO	**C/C**	9	0.692	62	0.775	11	0.524	0.65 (0.18-2.37)	0.498	2.04 (0.48-8.77	0.477
	**A/C**	4	0.308	16	0.200	10	0.476	1.78 (0.48-6.52)	0.467	0.49 (0.12-2.10)	0.477
rs577912	**A/A**	0	---	2	0.025	0	---	---	---	---	
	**A/***	4	0.308	18	0.225	10	0.476	1.53 (0.42-5.56)	0.498	0.49 (0.12-2.10)	0.477
	**C/C**	4	0.308	30	0.375	9	0.429	0.74 (0.21-2.62)	0.762	0.59 (0.27-1.86)	0.718
	**C/T**	7	0.538	38	0.475	10	0.476	1.29 (0.40-4.18)	0.769	1.26 (0.38-5.13)	0.999
rs564481	**T/T**	2	0.154	12	0.150	2	0.095	1.03 (0.20-5.24)	1.00	1.70 (0.26-14.09)	1.00
	**T/***	9	0.692	50	0.625	12	0.571	1.35 (0.38-4.77)	0.762	1.69 (0.39-7.27)	0.718
	**T/T**	8	0.615	55	0.688	13	0.619	0.73 (0.22-2.45)	0.750	0.98 (0.23-4.08)	1.00
	**G/T**	5	0.385	23	0.287	6	0.286	1.55 (0.46-5.24)	0.522	1.53 (0.33-6.76)	0.709
rs953614	**G/G**	0	---	2	0.025	2	0.095	---	---	---	---
	**G/***	5	0.385	25	0.313	8	0.381	1.37 (0.41-4.63)	0.750	1.02 (0.24-4.21)	1.00
FGF23	**G/G**	12	0.923	65	0.812	19	0.905	2.77 (0.33-22.99)	0.454	1.26 (0.10-15.49)	1.00
	**A/G**	1	0.077	13	0.163	1	0.048	0.43 (0.05-3.59)	0.683	2.32 (0.06-29.17)	1.00
rs7955866	**A/A**	0	---	2	0.025	1	0.048	---	---	---	---
	**A/***	1	0.077	15	0.188	2	0.095	0.36 (0.04-2.99)	0.454	0.79 (0.06-9.71)	1.00
IGF	**C/C**	10	0.769	54	0.675	14	0.666	1.61 (0.41-6.33)	0.748	1.67 (0.34-8.07)	0.704
	**C/T**	2	0.154	20	0.250	6	0.286	0.54 (0.11-2.67)	0.726	0.38 (0.06-2.66)	0.444
rs35767	**T/T**	1	0.077	6	0.075	1	0.048	1.03 (0.11-9.31)	1.00	1.66 (0.09-29.13)	1.00
	**T/***	3	0.231	26	0.325	7	0.334	0.62 (0.16-2.46)	0.748	0.60 (0.12-2.91)	0.704
TNF	**G/G**	12	0.923	73	0.912	17	0.810	1.15 (0.13-10.21)	1.00	2.82 (0.28-28.51)	0.627
	**A/G**	1	0.077	5	0.063	4	0.190	1.25 (0.13-11.65)	1.00	0.35 (0.06-3.58)	0.627
rs1800629	**A/A**	0	---	2	0.025	0	---	---	---	---	---
	**A/***	1	0.077	7	0.088	4	0.190	0.87 (0.10-7.71)	1.00	0.35 (0.06-3.58)	0.627
IL6	**G/G**	8	0.615	57	0.712	17	0.810	0.65 (0.19-2.18)	0.522	0.38 (0.08-1.79)	0.254
	**C/G**	5	0.385	17	0.213	4	0.190	2.32 (0.67-7.99)	0.180	2.68 (0.60-12.59)	0.254
rs1800795	**C/C**	0	---	6	0.075	0	---	---	---	---	
	**C/***	5	0.385	23	0.288	4	0.190	1.55 (0.46-5.24)	0.522	2.68 (0.60-12.59)	0.254
MIF	**G/G**	4	0.308	38	0.475	16	0.762	0.49 (0.14-1.73)	0.370	0.14 (0.02-0.65)	0.014
	**C/G**	5	0.385	19	0.238	5	0.238	2.01 (0.59-6.87)	0.309	2.00 (0.44-8.98)	0.450
rs755622	**C/C**	4	0.308	23	0.287	0	---	1.10 (0.31-3.94)	1.00	---	---
	**C/***	9	0.692	42	0.525	5	0.238	2.04 (0.58-7.16)	0.370	7.20 (1.53-33.84)	0.014
	**T/T**	3	0.231	31	0.388	8	0.381	0.47 (0.12-1.86)	0.361	0.49 (0.10-2.32)	0.465
	**C/T**	8	0.615	31	0.388	6	0.286	2.53 (0.76-8.44)	0.141	3.94 (0.90-16.90)	0.080
rs1007888	**C/C**	2	0.154	18	0.225	7	0.333	0.63 (0.13-3.09)	0.727	0.36 (0.06-2.11)	0.427
	**C/***	10	0.769	49	0.613	13	0.619	2.11 (0.54-8.27)	0.361	2.05 (0.43-9.78)	0.465

A: HC ESRD Vs NoHC ESRD
B: HC ESRD Vs HC CTRL
